# Pao Pereira extract suppresses benign prostatic hyperplasia by inhibiting inflammation-associated NFκB signaling

**DOI:** 10.1186/s12906-020-02943-2

**Published:** 2020-05-16

**Authors:** Yu Dong, Jiakuan Liu, Zesheng Xue, Jingya Sun, Zhengnan Huang, Yifeng Jing, Bangmin Han, Bing Shen, Jun Yan, Ruimin Huang

**Affiliations:** 1 Shanghai University, Shanghai, China; 2 Shanghai Institute of Materia Medica, Chinese Academy of Sciences, 555 Zuchongzhi Road, Shanghai, 201203 China; 3 Model Animal Research Center of Nanjing University, Nanjing, Jiangsu China; 4 Department of Urology, Shanghai General Hospital, Shanghai Jiaotong University, 100 Haining Road, Shanghai, 200080 China; 5 Department of Laboratory Animal Science, Fudan University, 130 Dong’an Road, Shanghai, 200032 China; 6 MOE Key Laboratory of Model Animals for Disease Study, Model Animal Research Center of Nanjing University, Nanjing, Jiangsu China; 7 University of Chinese Academy of Sciences, Beijing, 100049 China

**Keywords:** Pao Pereira extract, BPH, NFκB, Inflammation, Extracellular matrix

## Abstract

**Background:**

Our previous study revealed the extract from the bark of an Amazonian tree Pao Pereira can suppress benign prostatic hyperplasia (BPH) in a rat model. Herein, we examined its inhibitory effects on human BPH cells and dissect its molecular mechanism.

**Methods:**

We applied Pao extract to human BPH epithelial BPH-1 and prostate myofibroblast WPMY-1 cells. Cell viability, apoptosis and immunoblotting were performed, followed by gene expression profiling and gene set enrichment analysis (GSEA) to detect the differentially expressed genes and signaling pathway induced by Pao extract. Human ex vivo BPH explant organ culture was also used to examine the effects of Pao extract on human BPH tissues.

**Results:**

Pao extract treatment inhibited viability and induced apoptosis in human BPH-1 and WPMY-1 cells. Gene expression profiling and the following validation indicated that the expression levels of pro-apoptotic genes (*eg. PCDC4*, *CHOP* and *FBXO32*) were induced by Pao extract in both two cell lines. GSEA further revealed that Pao extract treatment was negatively associated with the activation of NFκB signaling. Pao extract suppressed the transcriptional activity of NFκB and down-regulated its target genes involved in inflammation (*CXCL5*, *CXCL6* and *CXCL12*) and extracellular matrix (ECM) remodeling (*HAS2, TNC* and *MMP13*) in both cultured cells and human ex vivo BPH explants.

**Conclusion:**

In both BPH epithelial and stromal cells, Pao extract induces apoptosis by upregulating the pro-apoptotic genes and inhibiting the inflammation-associated NFκB signaling via reducing phosphorylation of NFκB subunit RelA. Our data suggest that Pao extract may be a promising phytotherapeutic agent for BPH.

## Background

Benign prostatic hyperplasia (BPH) is a non-malignant enlargement of the prostate gland that is common in older males. About 70% of men over 70 will develop BPH [1]. Due to the excessive proliferation of the epithelial and stromal cells in the transition zone and periurethral glands, the enlarged prostate ultimately induces lower urinary tract symptoms (LUTS) including urgency and difficulty of urination [2, 3]. Moderate-to-severe LUTS have significant influences on the patients’ quality of life. The etiology of BPH is multi-factorial, including sex hormones, smooth muscle and inflammation [4]. Currently, medications for BPH/LUTS primarily target sex hormone synthesis and relief of tension in smooth muscle. However, alpha-blockers and 5α-reductase inhibitors (5-ARIs) induce non-trivial adverse side effects including asthenia and ejaculatory dysfunction [5]. Hence, more effective agents with fewer side effects are needed.

Chronic inflammation has been implicated in BPH development, as manifested by the infiltration of immune cells, including activated T cells and macrophages into human BPH tissues [6]. It was reported that chronic prostatic inflammation was associated with a larger prostate volume and a higher International Prostate Symptom Score (IPSS) [7].

Pao pereira (*Geissospermum vellosii*) is an Amazon rainforest tree, and its bark extract is used to treat malaria, digestive disorders and cancers. Pao extract is rich in β-carboline alkaloids [8] and exhibited anti-proliferative activities against melanoma and glioblastoma, ovarian cancer and pancreatic cancer [9–13]. Our previous study showed that Pao extract decreased human prostate cancer cell growth via inducing apoptosis [14]. Recently we found that Pao extract can attenuate BPH development in a rat model [15]. However, the molecular mechanism remains largely unclear.

In this study, we investigated the effects of Pao extract on human BPH epithelial cells (BPH-1) and stromal cells (WPMY-1). We found that Pao extract suppressed the growth of BPH-1 and WPMY-1 cells, and induced apoptosis via inhibition of NFκB signaling pathway. An ex vivo human BPH explant was also exploited to test the effects of Pao extract. These findings suggest that Pao extract may be a very promising therapeutic agent for BPH.

## Methods

### Cell lines, human BPH tissues and reagents

Human BPH-derived prostate epithelial BPH-1 cell line was kindly provided by Dr. Simon Hayward (Vanderbilt University Medical Center, Nashville, TN, USA). Human prostate myofibroblast WPMY-1 cell line was purchased from the Cell Bank of Type Culture Collection of Chinese Academy of Sciences (Shanghai, China). BPH-1 and WPMY-1 cells were cultured in RPMI 1640 (Corning, New York, NY, USA) and DMEM medium (Corning), respectively, containing 10% fetal bovine serum (FBS; Life Technologies, Carlsbad, CA, USA) and penicillin-streptomycin (S110JV, BasalMedia, Shanghai, China). Passage number of BPH-1 and WPMY-1 cell lines was less than 20 for all the cell-based experiments. The study protocol using human BPH tissues was approved by the Ethics Committee of Shanghai General Hospital, Shanghai Jiaotong University. Human BPH tissues were collected also with patients’ consent. Pao extract was from Natural Source International (New York, NY, USA). Briefly, the extract was prepared with aqueous alcoholic extraction of the bark of the plant Pao Pereira, which was then transformed into a free-flowing powder by spray drying. It contained 54% β-carboline alkaloids, including flavopereirine, by high-performance liquid chromatography [13, 15]. The Pao extract was dissolved in DMSO and diluted with sterile phosphate buffered saline (PBS) to 50 mg/ml as a stock solution and stored at − 80 °C until use. The final concentration of DMSO was less than 6% (v/v).

### Antibodies

Primary antibodies targeting the following proteins were used: Caspase-3 (#9662, Cell Signaling Technology (CST), Danvers, MA, USA, 1:1000), Cleaved Caspase-3 (#9661, CST, 1:1000), PARP (#9532, CST, 1:1000), Cleaved PARP (#5625, CST, 1:1000), NFκB/p65 (#8242, CST, 1:1000), Phospho-NFκB/p65(Ser536) (#3033, CST, 1:1000), PDCD4 (12587–1-AP, Proteintech, Rosemont, IL, USA, 1:1000), β-Actin (A2228, Sigma-Aldrich, St Louis, MO, USA, 1:5000), and CHOP (#2895, CST, 1:1000). Peroxidase AffiniPure goat anti-mouse IgG (H + L) (115–035-003, 1:2500) and peroxidase AffiniPure goat anti-rabbit IgG (H + L) (111–035-003, 1:2500) were purchased from Jackson ImmunoResearch (West Grove, PA, USA) and used as the secondary antibodies.

### Cell cytotoxicity assay

BPH-1 cells (1.5 × 10^3^ cells/well) and WPMY-1 cells (2.5 × 10^3^ cells/well) were seeded into 96-well plates in triplicate. The next day Pao extract with different concentrations was added into culture medium. 24, 48 and 72 h later, the cells were fixed with 10% trichloroacetic acid (TCA; Sigma-Aldrich) over 4 h and stained with 4 mg/ml sulforhodamine B (SRB; Sigma-Aldrich) in 1% acetic acid. 10 mM Tris base solution was used to dissolve the protein-bound dye. Optical density (OD) at 560 nm was determined by a microplate reader (SpectraMax M5, Molecular Devices, Sunnyvale, CA, USA).

### Apoptosis assay

Apoptosis assay was performed using FITC Annexin V Apoptosis Detection Kit I (#556547, BD Biosciences, Franklin Lakes, NJ, USA). BPH-1 and WPMY-1 cells were seeded into 6-well plates at densities of 5 × 10^4^ cells/well and 1 × 10^5^ cells/well, respectively. The next day, the medium was replaced with fresh medium and the cells were treated with Pao extract or cisplatin (5 μM, a positive control) for 72 h. Then the floating and adherent cells were collected, washed with PBS twice and re-suspended in 1 × Binding Buffer as 1 × 10^6^ cells/ml. 100 μl cell suspension was stained with 5 μl of FITC-Annexin V and 5 μl propidium iodide (PI) for 15 min at room temperature in the dark. Flow cytometry analysis was performed with a FACS Calibur flow cytometer (BD Biosciences) and cell death patterns were quantified (Figure S1) with FlowJo software (version 10.0.7r2, Ashland, OR, USA).

### Gene expression profiling analysis

BPH-1 and WPMY-1 cells were treated with Pao extract for 24 h, and total RNA was extracted with TRIzol reagent (Life Technologies). Affymetrix GeneChips (Human Transcriptome Array 2.0) were used for the gene expression profiling analysis (Shanghai Baygene Biotechnologies, Shanghai, China). The raw and normalized microarray data from this study can be accessed at GSE128856 in NCBI GEO Datasets. Gene Set Enrichment Analysis (GSEA) was performed using the GSEA software (v3.0, http://software.broadinstitute.org/gsea/index.jsp) [16] to determine the association between the priori defined gene sets in the Molecular Signatures Database (MSigDB) and the different changes of genes induced by Pao extract treatment. The number of permutations was 1000. Enrichment statistic was weighted and the ranking metric was the difference of class means (Diff_of_Classes). Normalized enrichment score (NES) ≥ 1 and the false discovery rate (FDR) < 0.25 were used as the cutoff.

### Western blotting

Cells were lysed with 2% SDS lysis buffer and total protein was quantified with the Enhanced BCA Protein Assay Kit (P0009, Beyotime, Shanghai, China). 10 ~ 20 μg total protein was separated on an SDS-PAGE gel and transferred onto a polyvinylidene difluoride (PVDF) membrane (Millipore, Billerica, MA, USA). The membranes were blocked with 5% bovine serum albumin (BSA; FA016, Gen-view Scientific, Houston, TX, USA) and incubated with the primary antibodies overnight at 4 °C. Then the membranes were incubated with horseradish peroxidase (HRP)-conjugated secondary antibodies for 1 h at room temperature. The signals were developed with SuperSignal West Pico PLUS Chemiluminescent Substrate (Thermo Fisher Scientific, Waltham, MA, USA) and detected by the Mini Chemiluminescent Imaging and Analysis System (Beijing Sage Creation Science, Beijing, China).

### RNA isolation and quantitative real-time PCR

The total RNA from cells was extracted using TRIzol, according to the manufacturer’s instructions. RNA was dissolved in DEPC-treated water (Sangon Biotech, Shanghai, China). The concentration and purity of RNA were measured with a NanoDrop One Microvolume UV-Vis Spectrophotometer (Thermo Fisher Scientific), and RNA was kept at − 80 °C. Residual DNAs in total RNAs were removed and cDNAs were synthesized by Hifair II 1st Strand cDNA Synthesis SuperMix (11123ES60, YEASEN, Shanghai, China) following the manufacturer’s protocol. Quantitative real-time PCR was performed by ChamQ Universal SYBR qPCR Master Mix (Q711–02, Vazyme, Nanjing, China) using a CFX96 Touch Real-Time PCR Detection System (Bio-Rad, Hercules, CA, USA) with the reaction conditions: Stage 1, 95 °C for 30 s; Stage 2, 40 cycles of 95 °C for 10 s and 60 °C for 30 s; Stage 3, 95 °C for 15 s, 60 °C for 60 s and 95 °C for 15 s. Data analysis was performed using the ΔΔCt method. Fold change was determined in relative quantification units using *ACTB* gene for normalization. Primers were listed in Table S1.

### Dual-luciferase reporter gene assay

BPH-1 and WPMY-1 cells were seeded into 24-well plates (5 × 10^4^ cells/well). 200 ng 6 × NFκB-Luc plasmid and 50 ng pRL-CMV plasmid for each well were transfected into the cells at ~ 70% confluency by Lipofectamine 3000 (Invitrogen, Carlsbad, CA, USA) in the presence of FBS. Eight hours after transfection, Pao extract was added for 36 h (BPH-1 cells) and 40 h (WPMY-1 cells), respectively. Cell lysates were then measured by Dual-Luciferase Reporter Assay System (E1910, Promega, Madison, WI, USA). The ratio of firefly luciferase activity versus Renilla luciferase activity was determined for NFκB transcriptional activity.

### BPH ex vivo explant culture

Human BPH tissues (*n* = 4) were obtained from patients with a transurethral resection of the prostate at Shanghai General Hospital, Shanghai Jiaotong University. The BPH ex vivo explant culture was described previously [17, 18]. In brief, the BPH tissues were subdivided into 3–5 mm^3^ pieces and cultured on absorbable gelatin sponges (HSD-B, HUSHIDA, Nanchang, China) in 6-well plates containing 4 ml DMEM/F-12 (Corning) with 10% FBS, antibiotic /antimycotic solution (S120, BasalMedia), 0.01 mg/ml insulin (I1882, Sigma-Aldrich) and 0.01 mg/ml hydrocortisone (H0135, Sigma-Aldrich). Tissues were treated with Pao extract for 48 h at 37 °C. The tissues were washed with PBS twice and then grinded with the High Throughput Tissue Grinder (Scientz-48, Ningbo Scientz Biotechnology, Ningbo, China) in protein lysis buffer or TRIzol.

### Statistical analysis

Statistical analyses were performed using GraphPad Prism Software (version 8.0.1, GraphPad, San Diego, CA, USA). Two-tailed Student’s *t* test was used to compare the difference between two groups, and *P* < 0.05 was considered statistically significant.

## Results

### Pao extract inhibited proliferation of BPH-1 and WPMY-1 cells

**Fig. 1:**
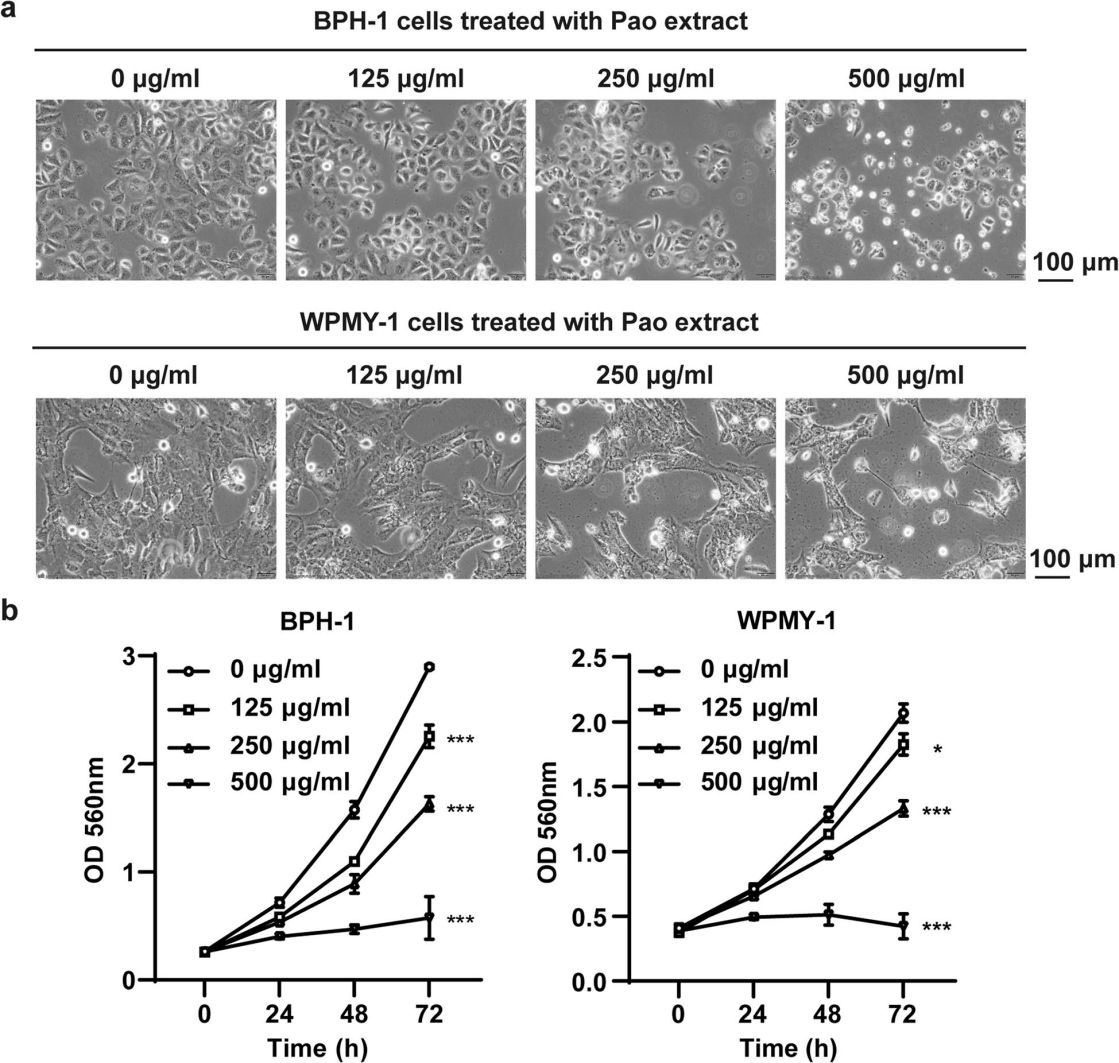
Pao extract inhibited the proliferation and induced apoptosis in BPH-1 and WPMY-1 cells. **a** Morphological changes of BPH-1 and WPMY-1 cells upon Pao extract treated for 48 h. **b** Effects of Pao extract on cell viability by the SRB assay. BPH-1 and WPMY-1 cells were treated with Pao extract at the indicated concentrations for 0, 24, 48 and 72 h. Data are presented as mean ± SD; * *p* < 0.05, ** *p* < 0.01, and *** *p* < 0.001

To evaluate the effect of Pao extract on the growth of cells derived from the prostate, BPH-1 and WPMY-1 cells were exposed to Pao extract with dosages ranging from 125 to 500 μg/ml, and the cell proliferation were assessed by SRB assay. Pao extract treatment significantly reduced the adherent cell number at 48 h (Fig. 1a) and viability within 72 h (*p* < 0.05) in a concentration-dependent manner in both cell lines (Fig. 1b).

### Pao extract induced apoptosis in BPH-1 and WPMY-1 cells

**Fig. 2:**
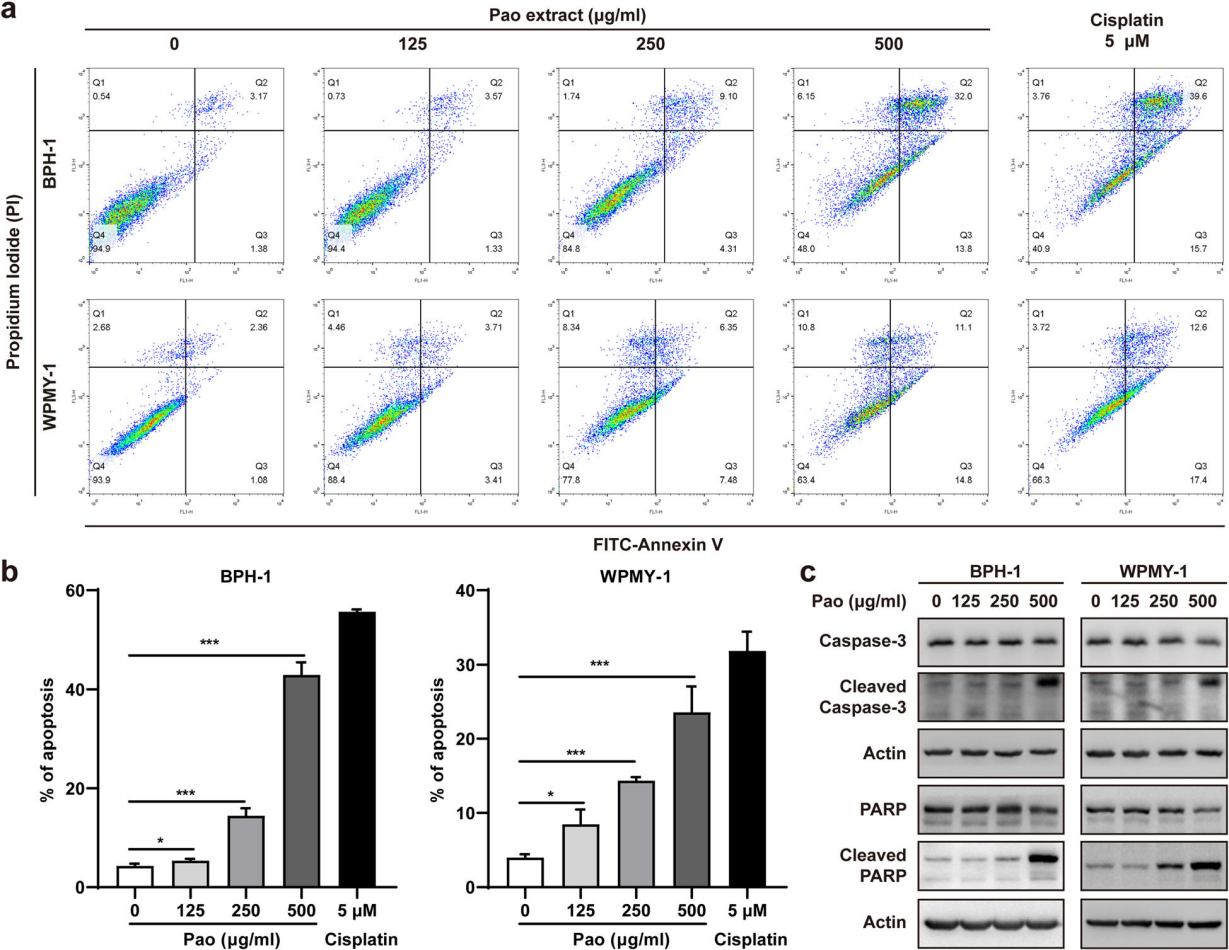
Pao extract induced apoptosis in BPH-1 and WPMY-1 cells. **a** Flow cytometry analysis on BPH-1 and WPMY-1 cells treated with Pao extract using an Annexin V-FITC/PI double staining kit. Representative plots were shown. **b** Quantification of cell death rates in BPH-1 and WPMY-1 cells treated with Pao extract. Annexin V-positive cells were defined as apoptotic cells. **c** Effects of Pao extract on the apoptosis-related proteins by Western blotting assay. Data are presented as mean ± SD; * *p* < 0.05, ** *p* < 0.01, and *** *p* < 0.001

Flow cytometry analyses were performed to determine whether Pao extract could induce apoptosis in BPH-1 and WPMY-1 cells. After 72 h treated with Pao extract, both cells showed that the percentages of apoptotic cells were significantly increased in a concentration-dependent manner by Annexin V-FITC/PI double staining (Fig. 2a). Notably, apoptosis was detected in ~ 43% BPH cells and ~ 24% WPMY-1 cells under the Pao extract treatment at 500 μg/ml (Fig. 2b). Moreover, the levels of cleaved Caspase-3 and cleaved PARP were consistently increased in the Pao extract-treated cells (Fig. 2c).

### Pao extract regulated the pro-apoptotic and inflammation-associated genes in BPH-1 and WPMY-1 cells

**Fig. 3:**
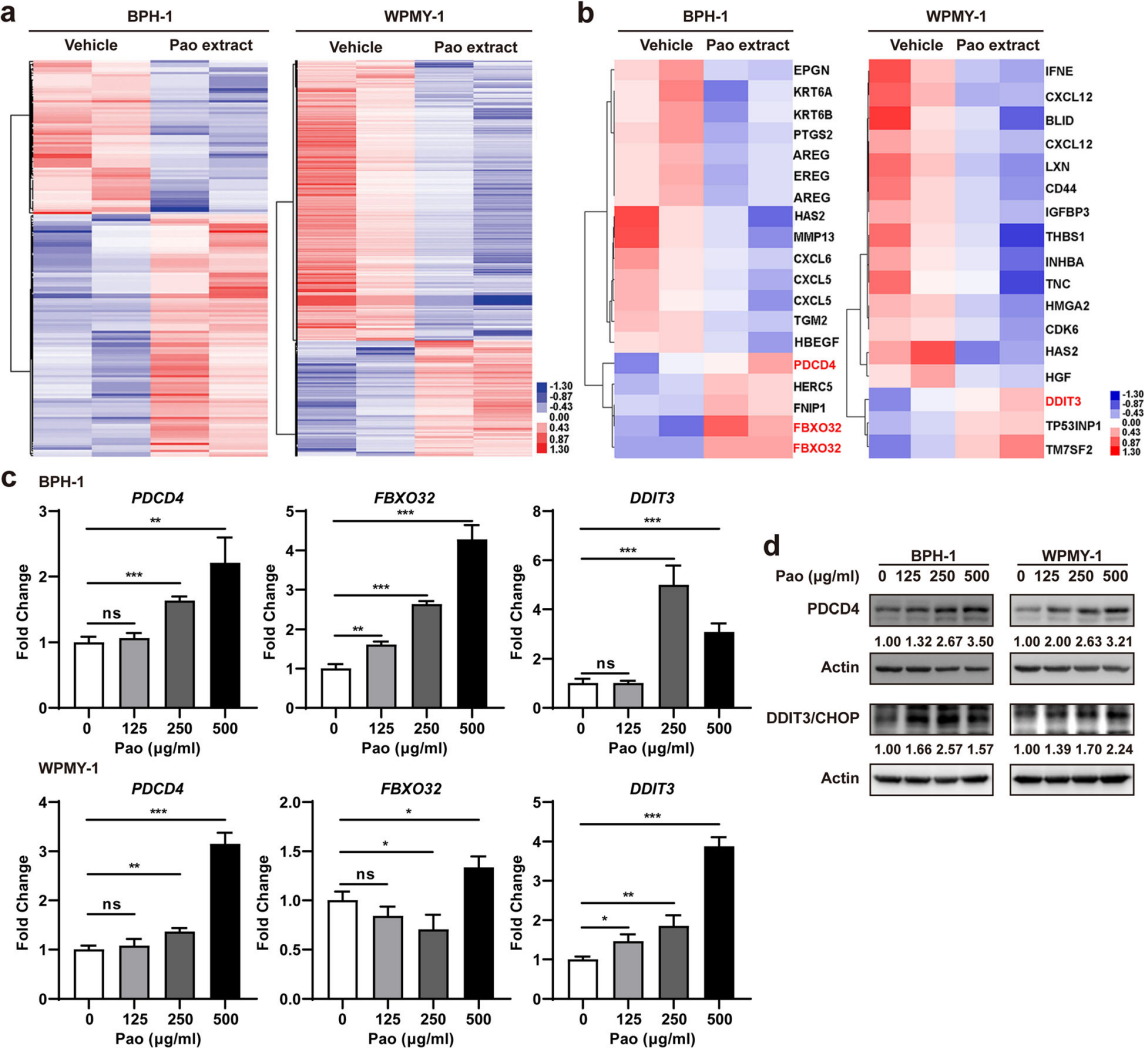
Pao extract regulated the pro-apoptotic and inflammation-associated genes in BPH-1 and WPMY-1 cells. **a** Downstream target genes of Pao extract detected by gene expression profiling analysis. BPH-1 and WPMY-1 cells were treated with Pao extract (250 μg/ml) or vehicle for 48 h. Heatmap of differentially expressed genes under the treatment of Pao extract by microarray (1.4-fold as the cutoff), compared with vehicle-treatment. Rows corresponded to genes and were ordered by hierarchical clustering. The values used for clustering were the expression levels normalized to average of all samples. **b** Heatmap of the pro-apoptotic and inflammation-associated genes from panel **a**. **c** The mRNA expression levels of pro-apoptotic and inflammation-associated genes (*PDCD4*, *DDIT3* and *FBXO32*) in BPH-1 and WPMY-1 cells by qRT-PCR. **d** The protein expression levels of pro-apoptotic and inflammation-associated genes (*PDCD4* and *DDIT3*) in BPH-1 and WPMY-1 cells by Western blotting. * *p* < 0.05, ** *p* < 0.01, and *** *p* < 0.001

To investigate which genes were regulated by Pao extract in both BPH-1 and WPMY-1 cells, gene expression profiling was performed using the Affymetrix microarray chips. Using 1.4-fold as the cutoff, 106 up-regulated genes and 68 down-regulated genes were identified in Pao extract-treated BPH-1 cells, comparing to the vehicle-treated cells (Fig. 3a, left panel); 212 up-regulated genes and 511 down-regulated genes were identified in Pao extract-treated WPMY-1 cells (Fig. 3a, right panel). Among them, several pro-apoptotic and inflammation-associated genes were induced by 250 μg/ml Pao extract treatment (Fig. 3b). We further confirmed the induction of the pro-apoptotic genes (*PDCD4*, *FBXO32* and *DDIT3*) in both BPH-1 and WPMY-1 cells by Pao extract at mRNA level (Fig. 3c). Western blotting data also indicated that Pao extract increases PDCD4 and DDIT3/CHOP at protein level (Fig. 3d).

### Pao extract inhibited NFκB signaling pathway in BPH-1 and WPMY-1 cells

**Fig. 4:**
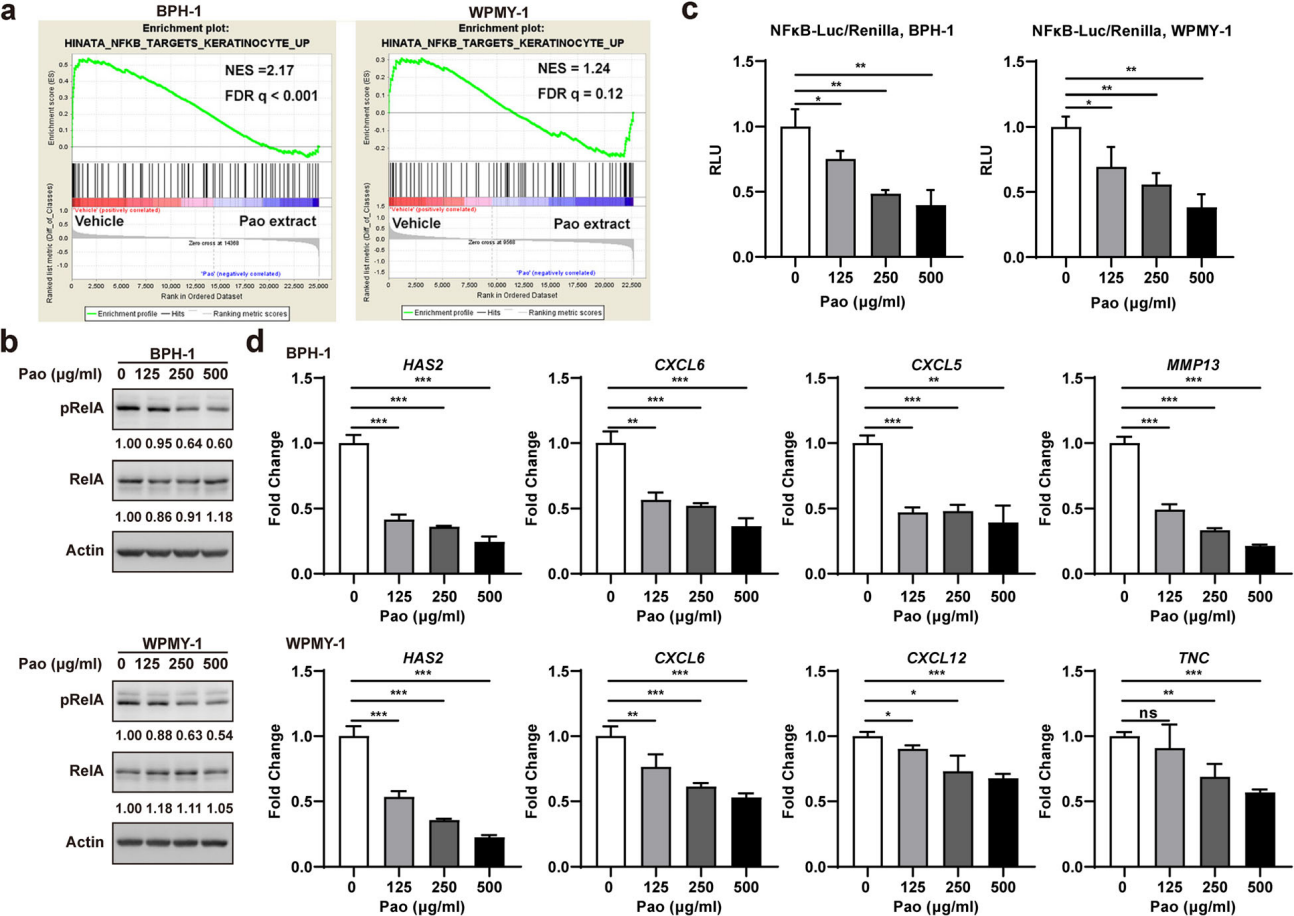
Pao extract inhibited NFκB signaling pathway in BPH-1 and WPMY-1 cells. **a** Gene set enrichment analysis identified the association between the down-regulation in gene set of NFκB signaling pathway and Pao extract treatment using microarray data from vehicle- and Pao extract-treated BPH-1 and WPMY-1 cells. In the enrichment plot, genes were ranked by signal/noise ratio according to their differential expression between vehicle- and Pao extract-treated cells. **b** Pao extract decreased phosphorylation of NFκB p65/RelA subunit in BPH-1 and WPMY-1 cells. **c** Pao extract inhibited transcriptional activities of NFκB after BPH-1 and WPMY-1 cells were treated with Pao for 36 h and 40 h, respectively. **d** Pao extract down-regulated the mRNA levels of NFκB target genes, including *CXCL5*, *CXCL6*, *CXCL12*, *HAS2*, *TNC*, and *MMP13* in BPH-1 and WPMY-1 cells by qRT-PCR. * *p* < 0.05, ** *p* < 0.01, *** *p* < 0.001, and ns *p* ≥ 0.05

We also used the aforementioned microarray data to investigate which signaling pathways were regulated by Pao extract in both BPH-1 and WPMY-1 cells by GSEA. The association between the down-regulation in gene set of NFκB signaling pathway and Pao extract treatment was identified in both BPH-1 cells (NES = 2.17, FDR < 0.001) and WPMY-1 cells (NES = 1.24, FDR = 0.12), respectively (Fig. 4a). The phosphorylation levels of RelA (a subunit NFκB transcription complex) were also shown to be reduced in BPH-1 and WPMY-1 cells by Pao extract using Western blotting (Fig. 4b). Moreover, NFκB-reporter was transiently transfected into BPH-1 and WPMY-1 cells, followed by Pao extract treatment. The luciferase activity from NFκB-reporter was suppressed by 60% in BPH-1 cells (*p* < 0.01) and by 62% in WPMY-1 cells (p < 0.01) using 500 μg/ml Pao extract (Fig. 4c). The expression levels of several well-known NFκB target genes involved in inflammation (*CXCL5*, *CXCL6*, and *CXCL12*) and extracellular matrix (ECM) remodeling (*HAS2*, *TNC*, and *MMP13*) were further tested. Consistently, *HAS2*, *CXCL6*, *CXCL5*, and *MMP13* were down-regulated in BPH-1 cells and *HAS2*, *CXCL6*, *CXCL12*, *TNC* were down-regulated in WPMY-1 cells by Pao extract (Fig. 4d). Altogether, it was suggested that Pao extract suppresses the activation of NFκB signaling in both BPH epithelial and stromal cells.

### Pao extract inhibited NFκB signaling pathway in human BPH tissues

**Fig. 5:**
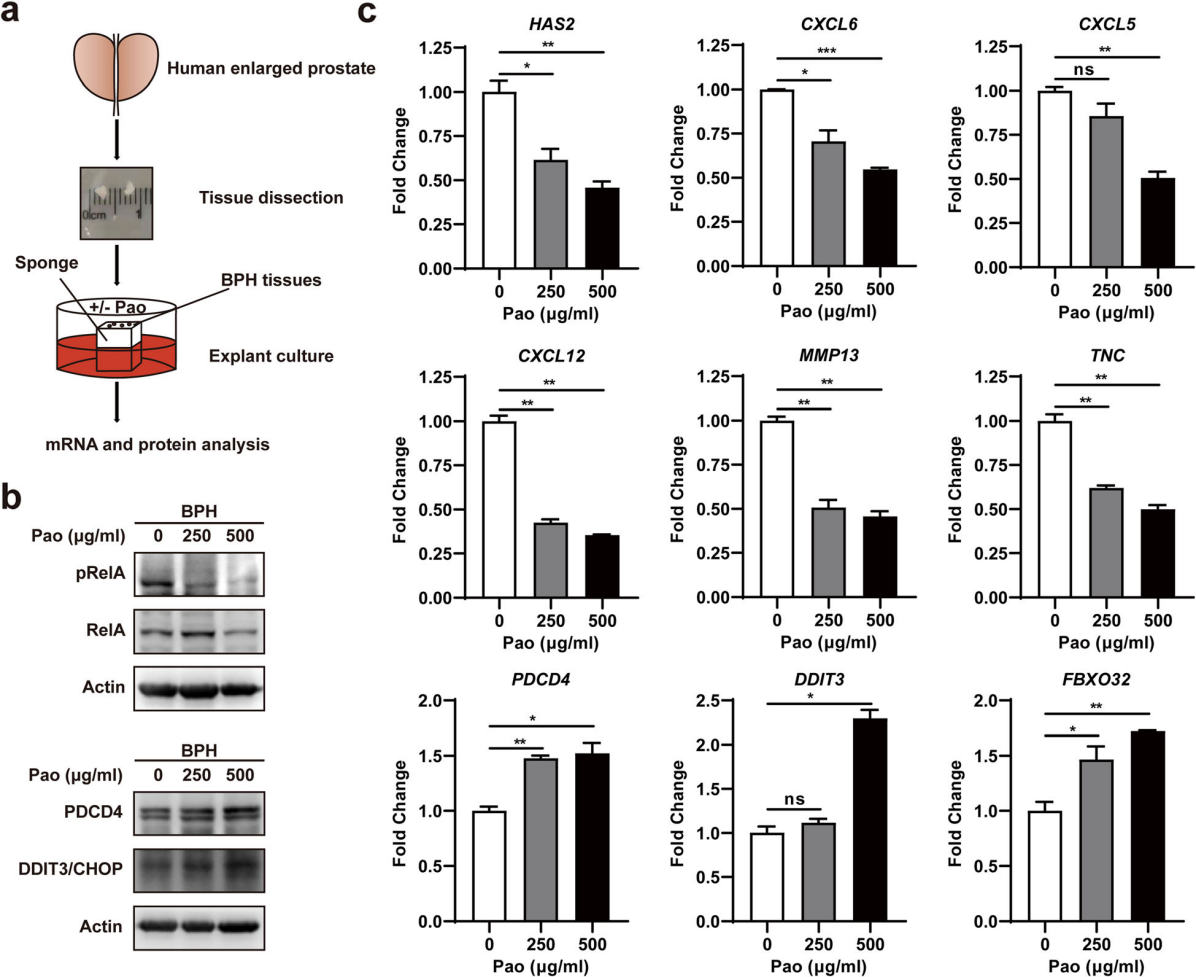
Pao extract inhibited NFκB signaling pathway in human BPH tissues. **a** Schematic diagram of the ex vivo BPH explant culture process. BPH explants were treated with 0, 250, 500 μg/ml Pao extract for 48 h. **b** The phosphorylation of RelA and protein expression levels of pro-apoptotic and inflammation-associated genes survival-associated proteins (PDCD4 and DDIT3/CHOP) were analyzed by Western blotting in BPH explants with Pao extract treatment. **c** The mRNA expression levels of target genes from NFκB signaling pathway, pro-apoptotic and inflammation-associated genes in BPH explants with Pao extract treatment by qRT-PCR. * *p* < 0.05, ** *p* < 0.01, *** *p* < 0.001, and ns *p* ≥ 0.05

To validate the effects of Pao extract on human BPH, an ex vivo explant culture system was established for the tissues from BPH patients (Fig. 5a). BPH explants were treated with 0, 250 and 500 μg/ml Pao extract for 48 h, following the assessment of NFκB signaling. Consistent with the data from cultured cell lines, the phosphorylation levels of RelA were markedly decreased and the protein levels of PDCD4 and DDIT3/CHOP were increased in Pao extract-treated BPH explants (Fig. 5b). In addition, the mRNA expression levels of the downstream target genes of NFκB signaling, including *CXCL6*, *HAS2*, *CXCL5*, *CXCL12*, *MMP13* and *TNC*, were also significantly reduced (*p* < 0.01), as well as the up-regulation of the apoptosis-associated genes *PDCD4*, *DDIT3* and *FBXO32* (*p* < 0.05), in BPH explants treated with Pao extract (Fig. 5c). Taken together, it was demonstrated that Pao extract inhibited the NFκB signaling pathway in human BPH tissues.

## Discussion

**Fig. 6:**
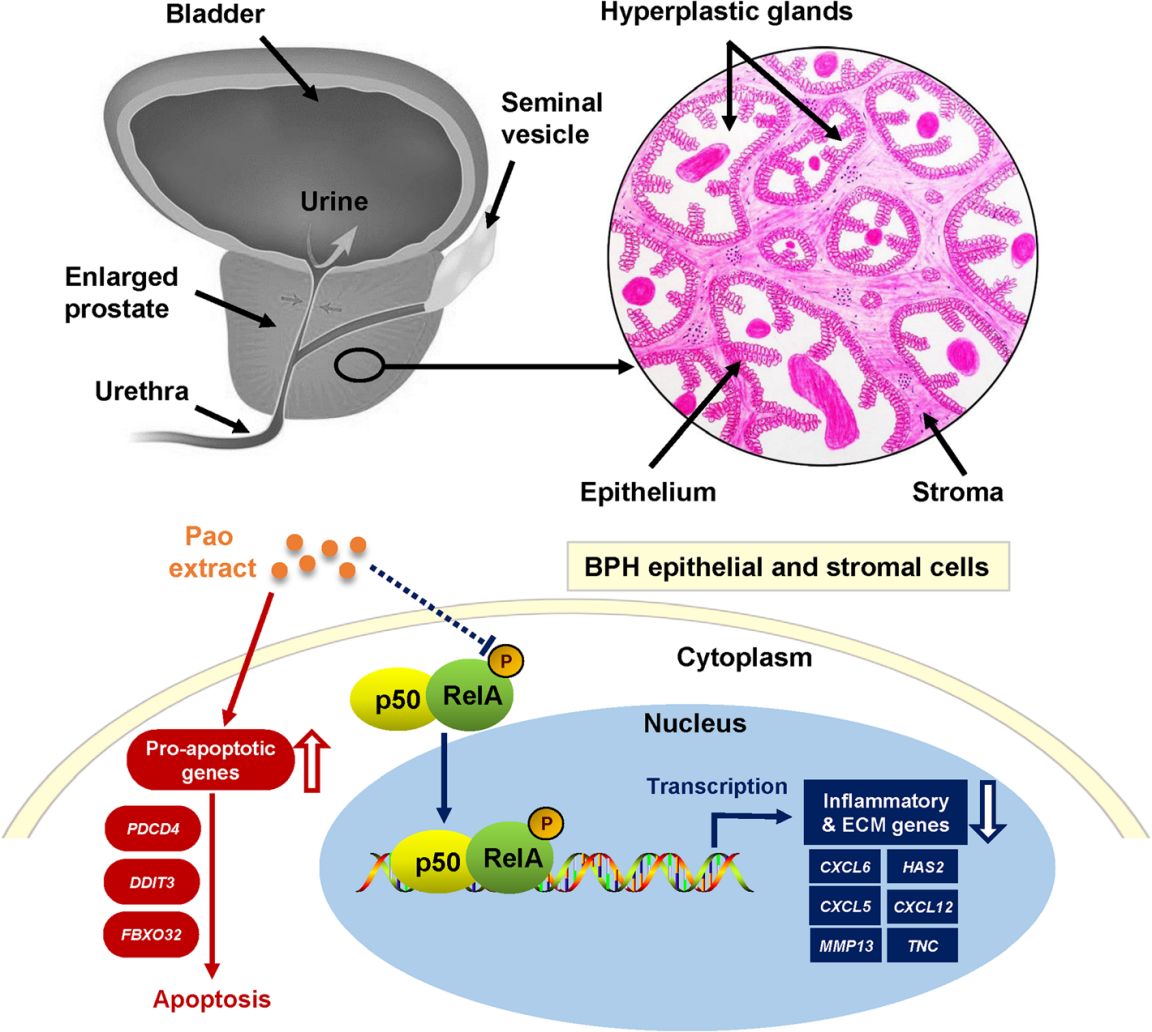
Schematic diagram of the working model for inhibitory effects of Pao extract on BPH tissues. BPH is characterized as the excessive proliferation of both epithelial and stromal cells in prostate. In both cell populations, Pao extract not only up-regulates the pro-apoptotic genes and induces apoptosis, but also inhibits the NFκB signaling and induces the inflammatory and ECM-associated genes expression via reducing phosphorylation of NFκB subunit RelA

Our previous study demonstrated that Pao extract suppresses testosterone-induced BPH in a rat model. To examine its effects on human BPH sample and delineate its molecular mechanism, herein we found that Pao extract inhibited the viabilities of BPH epithelial cells and stromal cells in a dose-dependent manner, due to the increase of apoptosis and suppression of NFκB signaling. Downregulation of inflammation- and ECM remodeling-related genes via NFκB signaling was further demonstrated in human BPH cell lines in vitro and in human BPH explants ex vivo (Fig. 6).

The canonical NFκB pathway is frequently up-regulated during the progression of BPH, and the severity of BPH is correlated with activation of NFκB [19], which makes it an interesting target for BPH. For example, Elocalcitol was reported to inhibit BPH stromal cells proliferation by targeting NFκB/p65 nuclear translocation [20]. Here, we showed that Pao extract could inhibit the phosphorylation level of RelA (Ser536), leading to the decrease of transcriptional activity of NFκB complex. In addition, Pao extract increased the mRNA and protein expression levels of two pro-apoptotic proteins, PDCD4 and DDIT3/CHOP. PDCD4 was reported to directly bind with NFκB/p65 and suppressed NFκB-dependent transcription in human glioblastoma [21]. Hence, it is suggested that Pao extract may target NFκB activity in BPH at different levels.

As an age-related disease, BPH progression is accompanied by chronic inflammation and ECM remodeling around BPH nodules. The inflammatory BPH microenvironment contains various secreted cytokines and chemokines, including CXCL5, CXCL6 and CXCL12, which are the direct NFκB target genes [22–24] and can promote the proliferation of both prostatic epithelial cells and stromal fibroblasts [25–27]. For the ECM-associated genes, such as *HAS2*, *MMP13* and *TNC*, they are also target genes of NFκB signaling. *HAS2* gene encodes hyaluronan synthase 2, an enzyme that synthesizes hyaluronan (HA) in BPH tissues [28, 29]. When BPH-1 cells were cultured in 3D gel containing collagen, they proliferated faster in the collagen from aged mice (high level of HA) than that from young mice (low level of HA). Previous studies also showed that MMP13 promoted ECM degradation, and the elevated ECM glycoprotein Tenascin-C was associated with myofibroblast in BPH tissues [30, 31]. Here, we observed that Pao extract downregulates the expression levels of *CXCL5, CXCL6*, *CXCL12*, *HAS2*, *MMP13* and *TNC* in BPH-1 and WPMY-1 cells, thus indicating that Pao extract attenuates the inflammation and ECM-remodeling via inhibition of NFκB signaling in BPH.

## Conclusions

Our data have proved the inhibitory effect of Pao extract on NFκB signaling pathway in two cell lines derived from human BPH and ex vivo explants from human BPH patients. Using Pao extract as a negative regulator of NFκB signaling may be a promising phytotherapeutic agent for BPH.

## Supplementary information

**Additional file 1: Figure S1.** Flow cytometry analysis on vehicle treated-BPH-1 cells (a) and WPMY-1 cells (b) stained with negative control buffer (Unstained), FITC-Annexin-V dye only (FITC only) and PI dye only (PI only). Representative plots were shown.

**Additional file 2: Table S1.** The sequences of primers used in quantitative real-time PCR assay.

## Data Availability

All microarray files are available from the NCBI GEO Datasets (accession number GSE128856). Other datasets used and/or analyzed during the current study are available from the corresponding author on reasonable request.

## Abbreviations

BPH: Benign prostatic hyperplasia
GSEA: Gene set enrichment analysis
LUTS: Lower urinary tract symptoms
5-ARIs: 5α-reductase inhibitors
IPSS: International Prostate Symptom Score
TCA: Trichloroacetic acid
SRB: Sulforhodamine B
OD: Optical density
PI: Propidium iodide
BSA: Bovine serum albumin
HRP: Horseradish peroxidase
ECM: Extracellular matrix
HA: Hyaluronan
