# Supplementary figures and images for: Pao Pereira extract suppresses benign prostatic hyperplasia by inhibiting inflammation-associated NFκB signaling
Source: BMC Complement Med Ther. 2020 May 16;20:150. doi: 10.1186/s12906-020-02943-2 (PMC7231430; doi:10.1186/s12906-020-02943-2)

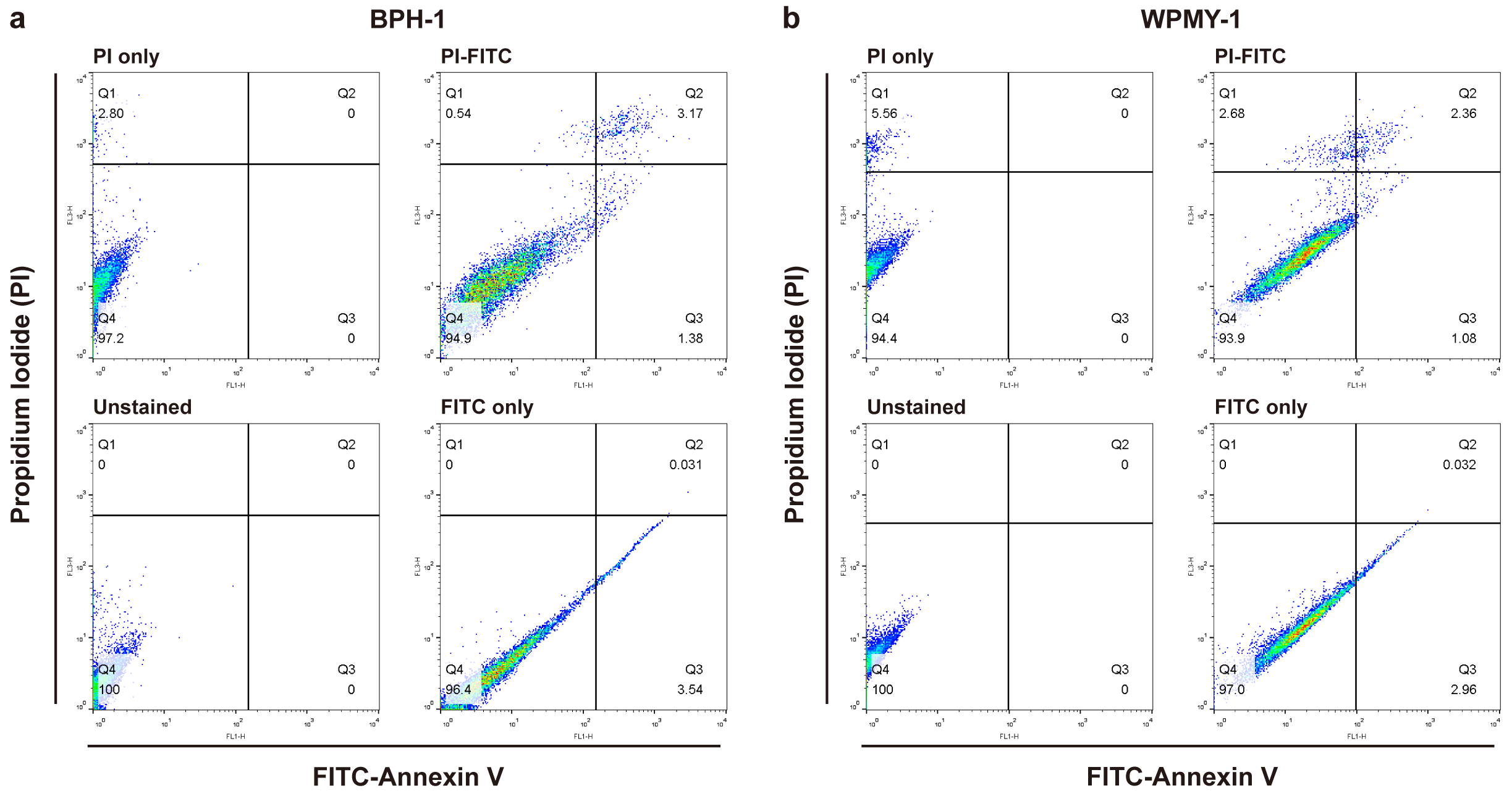

Supplement: Supplementary file 1 — Additional file 1: Figure S1. Flow cytometry analysis on vehicle treated-BPH-1 cells (a) and WPMY-1 cells (b) stained with negative control buffer (Unstained), FITC-Annexin-V dye only (FITC only) and PI dye only (PI only). Representative plots were shown. [file 12906_2020_2943_MOESM1_ESM.tif]
