# Supplementary material for: Pao Pereira extract suppresses benign prostatic hyperplasia by inhibiting inflammation-associated NFκB signaling
Source: BMC Complement Med Ther. 2020 May 16;20:150. doi: 10.1186/s12906-020-02943-2 (PMC7231430; doi:10.1186/s12906-020-02943-2)
Supplement: Supplementary file 2 — Additional file 2: Table S1. The sequences of primers used in quantitative real-time PCR assay. [file 12906_2020_2943_MOESM2_ESM.docx]

**Table S1. The sequences of primers used in qRT-PCR assay.**

| **Gene** | **Direction** | **Sequence (5’-3’)** |
| --- | --- | --- |
| *HAS2* | Forward | GTACACAGCCTTCAGAGCAC |
| *HAS2* | Reverse | TCTGTACATTCCCAGAGGTCCA |
| *ACTB* | Forward | CAGAGCCTCGCCTTTGCCGATC |
| *ACTB* | Reverse | CATCCATGGTGAGCTGGCGGCG |
| *CXCL6* | Forward | TGCGTTGCACTTGTTTACGC |
| *CXCL6* | Reverse | TCCAGAAAACTGCTCCGCTG |
| *CXCL5* | Forward | GAGAGCTGCGTTGCGTTTG |
| *CXCL5* | Reverse | TTTCCTTGTTTCCACCGTCCA |
| *CXCL12* | Forward | GCCAAGGTCGTGGTCGT |
| *CXCL12* | Reverse | CTTTAGCTTCGGGTCAATGCAC |
| *DDIT3* | Forward | CCTTTCTCCTTCGGGACACT |
| *DDIT3* | Reverse | CTCTGGGAGGTGCTTGTGAC |
| *FBXO32* | Forward | CTTCACTGACCTGCCTTTGTG |
| *FBXO32* | Reverse | TCCTTGGGTAACATCGGACAAG |
| *MMP13* | Forward | GCCATTACCAGTCTCCGAGG |
| *MMP13* | Reverse | TGTCATAACCATTAAGAGCCCA |
| *PDCD4* | Forward | TCTGGGAAAGGAAGGGGACT |
| *PDCD4* | Reverse | TGCCAACACTGGTACTCCAC |
| *TNC* | Forward | CCAAAGAGCCAACAAGCCAC |
| *TNC* | Reverse | AACGGTGTCTTCCAGAGCAG |
